# Standardized Endoscopic Swallowing Evaluation for Tracheostomy Decannulation in Critically Ill Neurologic Patients – a prospective evaluation

**DOI:** 10.1186/s42466-021-00124-1

**Published:** 2021-05-10

**Authors:** Paul Muhle, Sonja Suntrup-Krueger, Karoline Burkardt, Sriramya Lapa, Mao Ogawa, Inga Claus, Bendix Labeit, Sigrid Ahring, Stephan Oelenberg, Tobias Warnecke, Rainer Dziewas

**Affiliations:** 1 University Hospital Muenster, Department of Neurology with Institute for Translational Neurology, Albert-Schweitzer-Campus 1 A, 48149 Muenster, Germany; 2 Institute for Biomagnetism and Biosignalanalysis, University Hospital Muenster, Malmedyweg 15, 48149 Muenster, Germany; 3 Raphaelsklinik Muenster, Department of General Surgery, Loerstraße 23, 48143 Muenster, Germany; 4 University Hospital Frankfurt, Department of Neurology, Theodor-Stern-Kai 7, 60590 Frankfurt/Main, Germany; 5 Department of Rehabilitation Medicine I, School of Medicine, Fujita Health University, Toyoake, Aichi Japan; 6 Klinikum Osnabrück, Department of Neurology, Am Finkenhügel 1, 49076 Osnabrück, Germany

**Keywords:** Dysphagia, Tracheostomy, Decannulation, Intensive care, Aspiration, FEES

## Abstract

**Background:**

Removal of a tracheostomy tube in critically ill neurologic patients is a critical issue during intensive care treatment, particularly due to severe dysphagia and insufficient airway protection. The “Standardized Endoscopic Evaluation for Tracheostomy Decannulation in Critically Ill Neurologic Patients” (SESETD) is an objective measure of readiness for decannulation. This protocol includes the stepwise evaluation of secretion management, spontaneous swallowing, and laryngeal sensitivity during fiberoptic endoscopic evaluation of swallowing (FEES). Here, we first evaluated safety and secondly effectiveness of the protocol and sought to identify predictors of decannulation success and decannulation failure.

**Methods:**

A prospective observational study was conducted in the neurological intensive care unit at Münster University Hospital, Germany between January 2013 and December 2017. Three hundred and seventy-seven tracheostomized patients with an acute neurologic disease completely weaned from mechanical ventilation were included, all of whom were examined by FEES within 72 h from end of mechanical ventilation. Using regression analysis, predictors of successful decannulation, as well as decannulation failure were investigated.

**Results:**

Two hundred and twenty-seven patients (60.2%) could be decannulated during their stay according to the protocol, 59 of whom within 24 h from the initial FEES after completed weaning. 3.5% of patients had to be recannulated due to severe dysphagia or related complications. Prolonged mechanical ventilation showed to be a significant predictor of decannulation failure. Lower age was identified to be a significant predictor of early decannulation after end of weaning. Transforming the binary SESETD into a 4-point scale helped predicting decannulation success in patients not immediately ready for decannulation after the end of respiratory weaning (optimal cutoff ≥1; sensitivity: 64%, specifity: 66%).

**Conclusions:**

The SESETD showed to be a safe and efficient tool to evaluate readiness for decannulation in our patient collective of critically ill neurologic patients.

**Supplementary Information:**

The online version contains supplementary material available at 10.1186/s42466-021-00124-1.

## Background

Tracheostomy is a frequently performed procedure in the intensive care unit (ICU) [13, 49], with 10–15% of patients in polyvalent ICUs [1] and 15–46.8% in neurocritical care patients [21, 32]. In mechanically ventilated patients in the ICU – irrespective of the nature of the underlying disease – tracheostomy is used to prevent laryngeal and tracheal damage, to reduce the need for sedation, to shorten the duration of mechanical ventilation (MV), to increase patient comfort and to reduce the length of stay (LOS) in the ICU [13, 26, 33]. The prolonged presence of a tracheostomy tube (TT) can delay rehabilitation, cause complications, reduce patient comfort, and is associated with longer hospitalization and overall higher costs [8, 16, 20, 23]. Furthermore, it was shown that a TT in place at discharge from the ICU is predictive of a poor outcome [9, 29].

The removal of a TT, therefore, is a critical issue during intensive care and early rehabilitation. Severe dysphagia and insufficient airway protection are the main reasons for delayed decannulation and the patients need to remain tracheostomized [3]. Neurologic intensive care patients have a particularly high prevalence of oropharyngeal dysphagia (OD) [22, 35, 41] which makes this patient collective eminently prone to decannulation failure (DF). Decision making with regards to decannulation used to be largely based on the experience of the treating team of professionals. According to a recent systematic review, most studies use qualitative and quantitative determinants of coughing and swallowing to evaluate readiness for decannulation with swallowing mostly being subjectively assessed via gag reflex or Blue Dye Test [39]. To allow for an objective evaluation of decannulation safety in severely affected neurologic patients the “**S**tandardized **E**ndoscopic **S**wallowing **E**valuation for **T**racheostomy **D**ecannulation in Critically Ill Neurologic Patients” (SESETD) was introduced in 2013 [47]. This protocol includes a stepwise evaluation of secretion management, spontaneous swallowing and laryngeal sensation during flexible endoscopic evaluation of swallowing (FEES) at the bedside. In the original study, 54/100 consecutive neuro-ICU patients were decannulated based on this algorithm with one patient (1.9%) needing to be recannulated thereafter. Interestingly, this FEES-approach enabled decannulation nearly twice as often (54 vs. 29) compared to when the decision was based on the traditional clinical swallowing examination [24, 47]. Recently, inter-rater and test-retest reliability of the SESETD have been determined and it was shown that rating could reliably be performed by both experienced and inexperienced clinicians on every single item and the sum score [46]. In the meantime, the algorithm has been implemented in the guidelines of the French Intensive Care Society and the French Society of Anaesthesia and Intensive Care Medicine with a GRADE 2+ recommendation as possible examination at or as follow-up after decannulation [44] and was used as the primary endpoint in a recent interventional trial on tracheostomized stroke patients [14, 15].

In the present study, we provide data on the SESETD performance from 5 years’ experience in our neurologic ICU to assess the safety and effectiveness of this protocol in a larger patient cohort. Furthermore, we sought to add data, first on predictors of early decannulation and second on the need for recannulation.

## Methods

**Fig. 1:**
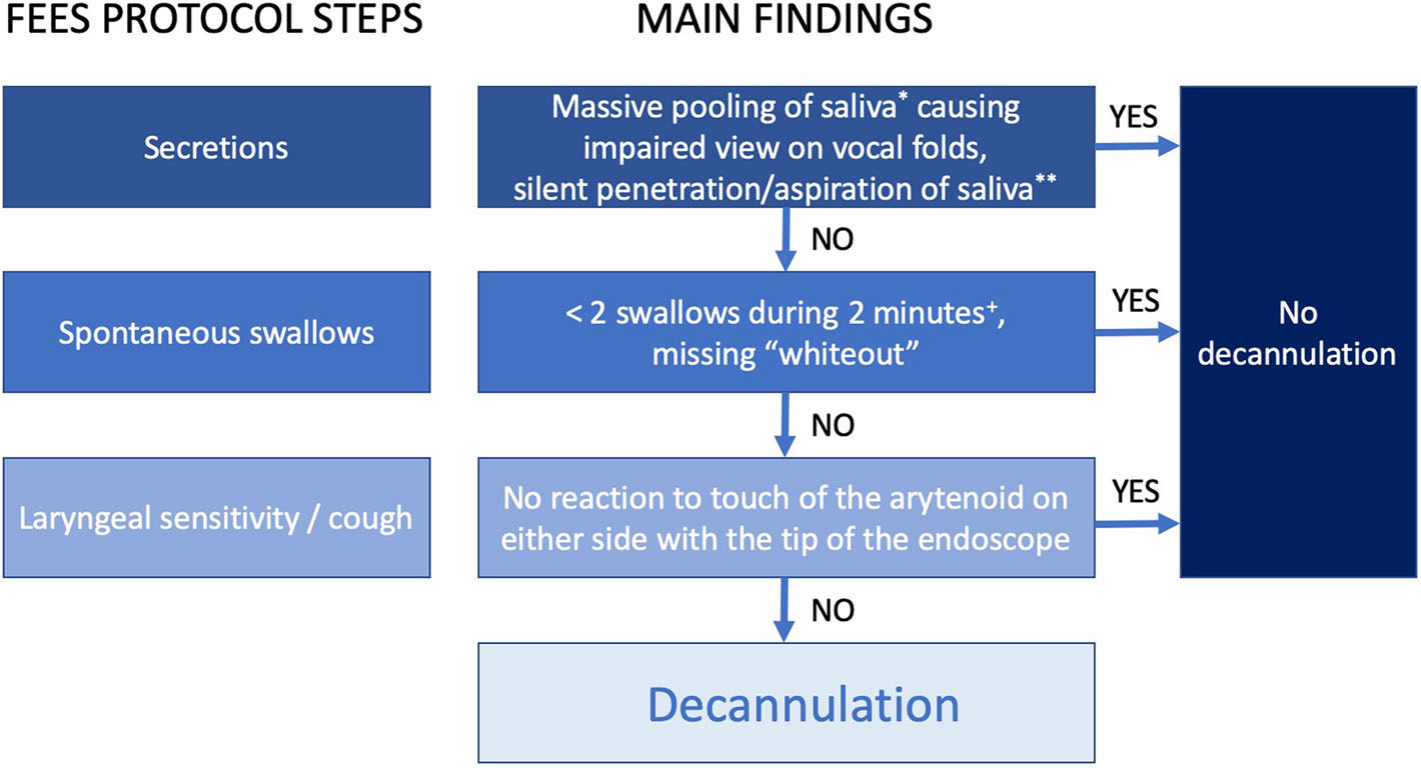
Stepwise evaluation of swallowing function according to the “Standardized endoscopic Swallowing Evaluation for tracheostomy decannulation in critically ill neurologic patients”; ^*^not only coating; ^**^permanently without any reaction; ^+^if exactly two swallows occur in this time period, another 2 minutes of observation are recommended (with permission from [18]: Warnecke T, Muhle P, Claus I, Schröder JB, Labeit B, Lapa S, Suntrup-Krueger S, Dziewas R. Neurol. Res. Pract. 2,9 (2020)

This prospective observational study was performed in the neurological ICU at Münster University Hospital. Between January 2013 and December 2017 consecutive patients with tracheostomy and FEES within 72 h after completion of weaning from MV according to the SESETD were included [47] (Fig. 1). Tracheostomy was performed due to the need for long-term ventilation and insufficient airway protection. If patients were considered not to be ready for decannulation (criteria see “Swallowing assessment” below), follow-up FEES were performed when clinical evaluation indicated improvement of swallowing function using the same protocol. All examinations were part of our local routine procedure for tracheostomy decannulation. None of the patients in this study were included in the prior study on this protocol [47]. This study was conducted in accordance with the amended Declaration of Helsinki. Data acquisition and analysis were approved by the local ethics committee and informed consent was given by the patients or their proxy if a patients’ communication was impaired.

### Baseline characteristics

Baseline characteristics, i.e. age, gender, neurological condition leading to admission and features of clinical treatment including reasons for intubation, duration of MV, LOS in the ICU and number and duration of anti-infective treatments were documented. Furthermore, pre-existing conditions likely to cause dysphagia, such as previous stroke, Parkinson’s disease, throat tumor, neoplasia other than throat tumor and/or dementia were systematically recorded based on the patients’ history. Also, the patients’ condition was assessed according to the following scores: the modified Rankin Scale (mRS; score from 0 being best to 6 meaning death) (on admission and discharge) to evaluate disability, the Richmond-Agitation-Sedation-Scale (RASS) to assess the degree of agitation and sedation, reaching from – 5 (= unarousable) to 0 (= alert and calm) to + 4 (combative) [38] and the Functional Oral Intake Scale (FOIS) to evaluate the functional oral intake (reaching from 1 = nil per mouth to total oral diet with no restrictions) [11] (each at discharge).

### Swallowing assessment

All swallowing assessments were performed by a trained neurologist and a speech-language pathologist with the patient being in an upright position (≥70°). Prior to FEES, the trachea was suctioned and the cuff deflated enabling the patient to breathe through the upper airway. The SESETD consists of a stepwise evaluation of three items: ‘secretion management’, ‘spontaneous swallows’ and ‘laryngeal sensibility/cough’, as previously published [14, 15, 46, 47]. Only if all three items are rated as passed, patients are considered ready for decannulation. Failure criteria for the item ‘saliva management’ are massive pooling (not only coating) causing an impaired view on the vocal folds and/or silent penetration and/or aspiration of pooled saliva (permanently without any reaction). The item ‘spontaneous swallows’ is considered failed if less than two swallows occur during 2 minutes of observation. If exactly two swallows occur in this period, another 2 minutes of observation is recommended. If no reaction to touch of the arytenoids with the tip of the endoscope on both sides can be elicited, the item laryngeal sensibility is considered as failed. For further analysis, the binary outcome of each item of the SESETD was transformed into a sum score (ordinal outcome) ranging from 0 (no item passed) to 3 (all items passed; ready for decannulation) [15, 37, 46]. Following this, we investigated whether the initial sum score is predictive of swallowing improvement during course of stay.

### Decannulation success and failure

The two major endpoints in this study were decannulation failure and decannulation success (early) during stay in the ICU. Decannulation was performed only if all three items of the SESETD were considered as passed. Early decannulation success was defined as removal of TT within 24 h from first FEES after completed weaning from MV. Decannulation was considered as failed if patients needed to be recannulated, respectively reintubated during their stay in the ICU due to dysphagia-related complications.

### Statistical analysis

Descriptive statistics were used to quantify baseline characteristics. Normal distribution was tested with the Kolmogorov-Smirnov test. If normal distribution was not given, the Mann-Whitney U test was used for comparison between the groups of those ‘decannulated’ and ‘not decannulated’. For comparison of multiple independent samples, the Kruskal-Wallis test was adopted. Post-hoc analysis for specific sample-pairs was performed with the Dunn-Bonferroni test. For dichotomized data, the Pearson chi-square test, respectively the Fisher exact test, if contingency tables contained less than 5 cases, were applied. Logistic regression analyses were used to identify factors predicting early decannulation success, decannulation success any time during the entire stay in the ICU, as well as decannulation failure including significant factors from the univariate analysis, as well as the easily available and clinically relevant factor age (per year) and duration of MV. The factor sum score of the SESETD at initial FEES after end of weaning was not included to predict early decannulation success, since the decision to decannulate depended on this score. Optimal cutoff values of the sum score were determined by receiver operator characteristics (ROC) analysis with maximizing the Youden Index. All analyses were carried out using SPSS 26.0 (IBM, Armonk, USA).

## Results

### Baseline characteristics

**Fig. 2:**
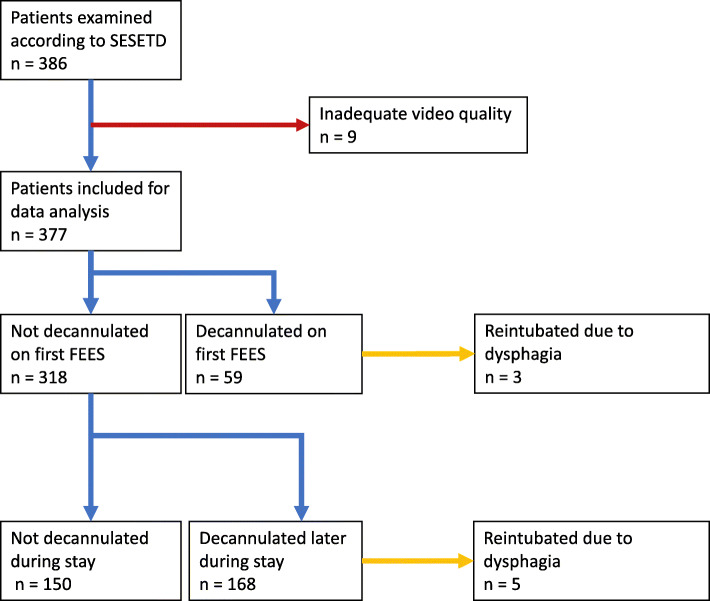
Study profile; SESETD = Standardized Endoscopic Swallowing Evaluation for Tracheostomy Decannulation in Critically Ill Neurologic Patients; FEES = Flexible Endoscopic Evaluation of Swallowing; GCS = Glasgow Coma Scale

From 2013 to 2017, 377 patients (158 females) fulfilled the inclusion criteria (Fig. 2). The mean age was 62.6 ± 15.7 yrs. Most patients suffered from ischemic (58.1%) or hemorrhagic stroke (17.2%). Guillain-Barré syndrome (4.0%), meningitis (8.2%) or myopathy (0.8%) were found less frequently as the primary diagnosis. Clinical features are presented in Table 1. Two hundred and twenty-seven patients (60.2%) were decannulated during their stay in the ICU, whereas 150 (49.8%) could not be decannulated according to the protocol’s criteria. Patients stayed in the ICU for 28.2 ± 18.9d after tracheostomy, respectively 11.0 ± 8.9d after decannulation. Comparing groups of patients who were decannulated to those who remained tracheotomized, the latter showed a worse mRS on admission (*p* = 0.037) and discharge (*p* < 0.001) and more often had a history of neoplasia other than ENT related tumors (*p* = 0.041). Patients suffering from intracranial hemorrhage were decannulated less frequently (*p* = 0.037). Patients who remained cannulated had a worse mRS (*p* < 0.001), FOIS (*p* < 0.001) and RASS at discharge (*p* < 0.001) and were dependent on PEG/nasogastric tube more often (52.9 vs. 90.7%) (*p* < 0.001). FEES could be performed safely in all participants.

**Table 1:** Basic patient characteristics and outcome parameters depending on variable decannulated / not decannulated (IQR = interquartile range; GBS = Guillain Barré syndrome; h = hours; d = days; mRS = modified Rankin Scale; GCS = Glasgow Coma Scale; LOS = length of stay; ICU = intensive care unit; FOIS = Functional Oral Intake Scale; RASS = Richmond Agitation and Sedation Scale); * Mann-Whitney U-test ** Chi^2^/ Fisher-exact test

Characteristic	TOTAL ***N*** = 377	PATIENTS DECANNULATED ***N*** = 227 (60.2%)	PATIENTS NOT DECANNULATED ***N*** = 150 (39.8%)	***P***
Age, MEDIAN (IQR)	65 (52–75)	65 (50–74)	65 (57–76)	0.06*
Female / Male, N (%)	158 (41.9)/219 (58.1)	101 (44.5) / 126 (55.5)	57 (38.0)/ 93 (62.0)	0.24**
Ischemic stroke (%)	221 (58.6)	136 (59.9)	86 (57.3)	0.67**
Intracranial hemorrhage (%)	65 (17.2)	31 (13.7)	33 (22.0)	0.04**
Meningitis/Encephalitis (%)	31 (8.2)	22 (9.7)	9 (6.0)	0.25**
GBS (%)	15 (4.0)	8 (3.5)	7 (4.7)	0.60**
Myopathy/Myasthenia/Myositis (%)	3 (0.8)	2 (0.9)	1 (0.6)	1.00**
Other (%)	42 (11.1)	29 (12.8)	14 (9.3)	0.33**
Comorbidities likely causing dysphagia, N(%)	114 (30.2)	65 (28.6)	49 (32.7)	0.42**
Stroke before (%)	81 (21.5)	47 (20.7)	34 (22.7)	0.70**
Parkinson Disease (%)	4 (1.1)	1 (0.4)	3 (2.0)	0.31**
Throat tumor (%)	7 (1.9)	3 (1.3)	4 (2.7)	0.44**
Other neoplasia (%)	28 (7.2)	11 (4.8)	16 (10.7)	0.04**
Dementia (%)	11 (2.9)	6 (2.6)	5 (3.3)	0.76**
mRS on admission, MEDIAN (IQR)	5 (4–5)	5 (4–5)	5 (4–5)	0.04*
Days of antiinfective treatment, N (%)	24.8 (± 14.7)	24.7 (± 14.7)	25.1 (± 14.7)	0.87*
Duration of mechanical ventilation (h)	464.7 (± 363.5)	443.1 (± 319.2)	497.4 (± 420.8)	0.74*
Decannulation within 24 h after 1. FEES, N (%)	59 (15.6)	59 (26.0)	–	–
Reintubation due to respiratory failure / dysphagia, N (%)	8 (2.1)	8 (3.5)	**–**	**–**
Sum Score SESETD First FEES after end of Weaning, mean	1.07 (± 1.22)	1.39 (± 1.27)	0.59 (0.98)	< 0.01*
Score 0, N (%)	180 (47.7)	81 (35.7)	99 (66.0)	
Score 1, N (%)	78 (20.7)	50 (22.0)	28 (18.7)	
Score 2, N (%)	31 (8.2)	23 (10.1)	8 (5.3)	
Score 3, N (%)	88 (23.3)	73 (32.2)	15 (10.0)	
LOS in the ICU (d) (SD)	37.3 (± 20.0)	36.4 (± 18.5)	38.7 (± 22.0)	0.64*
First FEES until discharge from ICU (d) (SD)	15.4 (±14.0)	15.1 (± 13.3)	16.0 (± 15.0)	0.71*
mRS at discharge, MEDIAN (IQR)	5 (4–5)	4 (4–5)	5 (4–5)	< 0.01*
FOIS AT DISCHARGE, MEDIAN (IQR)	2 (1–4)	3 (1.5–5)	1 (1–1)	< 0.01*
Nasogastric tube/PEG at discharge, N (%)	256 (67.9)	120 (52.9)	136 (90.7)	< 0.01**
Deceased, N (%)	15 (4.0)	6 (2.6)	9 (6.0)	0.11**
RASS at discharge, MEDIAN (IQR)	0 (0–0)	0 (0–0)	0 (0–0)	0.03*

### Decannulation failure

Eight patients had to be reintubated due to respiratory failure after passing all items of the SESETD and subsequent removal of the TT, indicating DF in 3.5% of patients. The mean duration from decannulation to orotracheal reintubation was 81.1 ± 61.4 h. Three patients were reintubated within 24 h after decannulation. Two patients were reintubated between 48 and 96 h, and 3 between 96 and 168 h (supplement Fig. 1). Additionally, 3 patients needed to be reintubated but were not considered as DF due to respiratory failure/dysphagia and were therefore excluded from further analysis. One of these patients suffered from secondary intracranial hemorrhage (1d after decannulation), one patient was reintubated after 5 d due to a newly occurred laryngeal edema and one patient showed tracheomalacia and was recannulated directly after decannulation. As inferred from logistic regression analysis (Table 2), the duration of MV remained as the only significant predictor of DF due to respiratory failure (*p* < 0.01).

**Table 2:** Logistic regression analysis for decannulation failure mRS = modified Rankin Scale; cat = categorical; Nagelkerke R^2^: 0.175

PARAMETERS	REGRESSION COEFFICIENT B	EXP(B) [95% confidence interval]	P-LEVEL
AGE	−0.02	0.98 [0.94–1.03]	0.45
mRS ON ADMISSION	−0.12	1.23 [0.40–3.19]	0.83
MECHANICAL VENTILATION (HOURS)	0.00	1.00 [1.00–1.00]	< 0.01
INTRACRANIAL HEMORRHAGE (cat.)	17.24	30,728,104.3 [0.00 –.]	1.00
HISTORY OF NEOPLASIA OTHER THAN THROAT (cat.)	−0.82	0.44 [0.41–4.71]	0.50
SESETD SUM SCORE ON INITIAL FEES AFTER END OF WEANING	0.32	1.37 [0.75–2.52]	0.31
CONSTANT	−21.15	0.00	1.00

### Predictors of decannulation success

As inferred from logistic regression analysis considering the variables age, mRS on admission, duration of MV (h), intracranial hemorrhage and neoplasia other than throat, only lower age presented to be a significant predictor of early decannulation (*p* = 0.03). Regarding decannulation anytime during stay in the ICU including the same factors and additionally the initial sum score of the SESETD for regression analysis, only the initial SESETD score showed to be a significant predictor (*p* < 0.01) (Tables 3 and 4).

**Table 3:** Logistic regression analysis for early decannulation (within 24 h from first FEES after end of mechanical ventilation) mRS = modified Rankin Scale, cat = categorical; Nagelkerke R^2^: 0.047

PARAMETERS	REGRESSION COEFFICIENT B	ODDS RATIO	P-LEVEL
AGE	−0.02	0.98 [0.96–1.00]	0.03
mRS ON ADMISSION	−0.33	0.80 [0.50–1.04]	0.08
MECHANICAL VENTILATION (HOURS)	0.00	1.00 [1.00–1.00]	0.70
INTRACRANIAL HEMORRHAGE (cat.)	0.35	1.42 [0.60–3.34]	0.42
NEOPLASIA OTHER THAN THROAT (cat.)	0.91	2.49 [0.56–11.09]	0.23
CONSTANT	−0.15	0.86	0.91

**Table 4:** Logistic regression analysis for decannulation during stay in the ICU mRS = modified Rankin Scale; cat = categorical; Nagelkerke R^2^: 0.17

PARAMETERS	REGRESSION COEFFICIENT B	ODDS RATIO	P-LEVEL
AGE	−0.01	0.99 [0.98–1.01]	0.27
mRS ON ADMISSION	−0.18	0.84 [0.61–1.16]	0.28
MECHANICAL VENTILATION (HOURS)	−0.00	1.00 [1.00–1.00]	0.09
INTRACRANIAL HEMORRHAGE (cat.)	0.34	1.40 [0.78–2.50]	0.26
HISTORY OF NEOPLASIA OTHER THAN THROAT (cat.)	0.66	1.94 [0.83–4.53]	0.13
SESETD SUM SCORE ON INITIAL FEES AFTER END OF WEANING	0.57	1.76 [1.43–2.17]	**< 0.01**
CONSTANT	0.56	1.75	0.60

### Decannulation success depending on the initial score of the SESETD

**Fig. 3:**
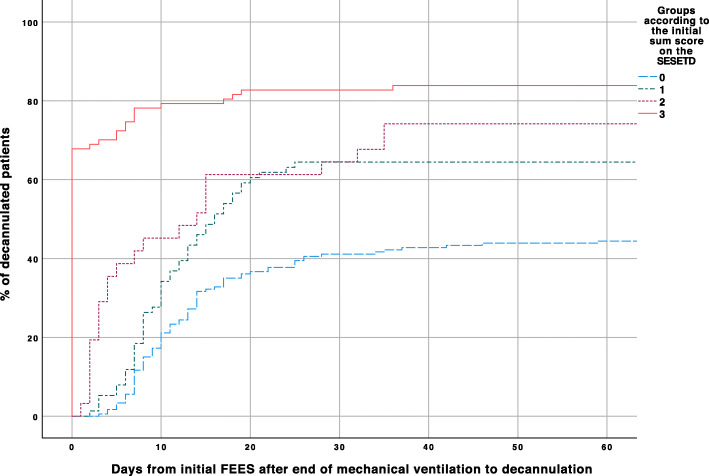
Distribution of decannulation according to the initial score on the SESETD (0; 1; 2; 3) and depending on the time between initial FEES and decannulation (days); SESETD = Standardized Endoscopic Swallowing Evaluation for Tracheostomy Decannulation in Critically Ill Neurologic Patients; Log Rank *p* < 0.000; Score 0: *n* = 180; Score 1: *n* = 78; Score 2: *n* = 31; Score 3: *n* = 88

The patient cohort was subdivided into four groups according to the initial SESETD score (0–3). Patients scoring higher early after end of weaning were more likely to be decannulated early, respectively at all during the stay in the ICU (Fig. 3). Patients failing all items initially were least likely to be decannulated at any time (score 0vs.1: *p* = 0.005**;** score 0vs.2: *p* = 0.003; score 0vs.3: *p* < 0.001). Sixty-seven percent of patients passing all items on the initial FEES could be decannulated immediately. The remaining 29 patients continued to be cannulated due to pneumonia (*n* = 7), laryngeal edema (*n* = 6), vocal cord paresis (*n* = 3), weak cough (n = 3), severe gastritis/esophagitis (*n* = 2), hypercapnia (*n* = 1), planned secondary intervention (n = 1) and reduced vigilance (≤8 points on the Glasgow Coma Scale) (*n* = 4). One patient suffered from amyotrophic lateral sclerosis and was not decannulated due to a poor prognosis. Of these 29 patients, another 14 could be decannulated during further treatment in the ICU, leaving 15 patients (17%) tracheostomized at discharge. Twenty-five percent of patients with an initial score 2 could be decannulated within the first 3d (25.8% remained cannulated), with a score 1 within 8d (35.9% remained cannulated) and with a score 0 within 13d (55% remained cannulated), respectively. Figure 2 of the supplement displays the results of the ROC analysis. The optimal cutoff to predict decannulation sometime during stay in the ICU was a sum score ≥ 1 (sensitivity: 0.64; specifity: 0.66; positive predictive value: 0.74; negative predictive value: 0.45).

## Discussion

In this prospective 5-year follow-up study, we assessed the safety and efficiency of the SESETD in a cohort of 377 neuro-critical care patients, as well as predictors of (early) decannulation and DF.

DF due to presumed dysphagia-related complications occurred in 3.5% of patients. This failure rate is in keeping with the suggestion of a recent cross-sectional survey. Here, the majority of 225 physicians and respiratory therapists regarded a recannulation rate of 2–5% as appropriate considering possible risks and benefits of TT removal [42]. Thus, on the one hand, reintubation/recannulation is associated with an increased risk for immediate procedure-related or early complications, i.e. hemorrhage, pneumothorax, infection, subcutaneous emphysema or hypoxia [12, 17]. While these issues seem to argue for a cautious approach minimizing the risk of DF, an unnecessarily prolonged cannulation is also associated with adverse outcomes. With longer periods of cannulation there is an increased risk of late complications, in particular tracheal stenosis, bleeding, fistulas, infections, aspiration as well as psychosocial side-effects [17, 18]. Additional consequences are longer hospitalization, delayed rehabilitation and overall higher costs [8, 16, 20, 23]. Therefore, any decannulation algorithm needs to find a reasonable balance between an aggressive approach targeting very early decannulation at the expense of higher rates of recannulation and a more conservative procedure trying to minimize recannulation risk while accepting longer cannulation times.

There cannot be a one-size-fits-all solution to this problem since both the specific case-mix and the medical environment impact on the choice of a specific strategy. Hence, it is not surprising that protocols, measures, and outcomes differ notably between most studies on decannulation safety. Investigations that use a stepwise downsizing of the TT, intermediate capping, cuff deflation, a fenestrated tube or patient-specific clinical parameters to evaluate decannulation show very heterogeneous results with failure rates from 0 to 77% [2, 5, 6, 8, 19, 27, 31, 34, 42].

Also, patient collectives differ distinctly between investigations. Bach and Saporito report failure rates of 32.4% on initial decannulation attempts in patients suffering from neuromuscular disorder [2], whereas in an acute setting, Ceriana et al. identified DF in 3.5% of patients in a mixed collective with patients mainly suffering from cardiopulmonary failure [6]. Studies that adopt instrumental evaluation of swallowing tend to have lower recannulation rates (1.9–20%) with some evidence that immediate decannulation leads to shorter hospitalization at similar safety compared to the gradual decrease of tube size and intermittent tube capping [10, 47]. With the need for reintubation in 3.5% the SESETD allows for a moderately aggressive approach for decannulation in patients with a particularly high risk of dysphagia in the acute care setting.

In our study, the duration of MV was a significant predictor of DF. Similarly, Budweiser et al. described longer periods of cannulation to be linked with DF in a mixed collective consisting mainly of patients with cardiopulmonary failure [5]. Bach and Saporito identified peak cough flow ≤160 l/min as a significant predictor of DF [2], Guerlain et al. described peak inspiratory flow < 40 l/min to be associated with DF in patients after head and neck cancer surgery [19] and Choate et al. found the inability to expectorate secretions to be a major reason for DF in a mixed collective [8]. Reduced peak cough and inspiratory flow, as well as the inability to expectorate secretions indicate muscular insufficiency, possibly as a result of critical illness neuromyopathy [25]. Occurrence and degree of critical illness neuromyopathy are related to the length of ICU treatment [45], thus, also explaining the higher likelihood of DF in patients with prolonged MV in the collective investigated by Budweiser et al. [5] and the one presented here. Other risk factors for decannulation failure are conceivable but have not been investigated so far. With a growing number of patients being multimorbid, further studies are needed to understand the role of reciprocal worsening of conditions with regard to weaning and decannulation decisions (e.g. a combination of stroke and COPD).

Concerning clinical risk management in recently decannulated patients, the period from decannulation to the occurrence of respiratory failure and subsequent reintubation requires a closer evaluation. In our study, the need for reintubation appeared mainly within the first few days after tube removal (3.4 ± 2.6d) during an observational period of 11 ± 9d, yet 3 patients needed reintubation later than day 4 after TT removal. Choate et al. reported DF to occur within 24 h from decannulation in 60% of cases and another 12.5% within the following 24 h in their collective mainly consisting of trauma patients [8]. Similarly, DF occurred in the majority of cases within 24-72 h in other studies [2, 7, 19, 47]. Definitions of DF differ vastly between studies, e.g. Guerlain et al. considered the need for recannulation within 24 h after decannulation as DF [19], whereas Choate et al. included patients who failed TT removal even after more than 1 week [8]. In the aforementioned cross-sectional survey by Stelfox et al., decannulation was considered as failed if it occurred within 48 h after TT removal by most experts [42]. Following this definition, our DF rate would have been 1.3%.

Our study provides further evidence that in the neuro-critically ill recovery of swallowing function may require significantly more time than respiratory weaning. In line with this, in the DECAST study, only 26% of patients acquired brain injury could be decannulated within 3 months after tracheostomy [36], whereas in the SETPOINT study 47% of patients with severe ischemic or hemorrhagic stroke were decannulated during the observational period of 6.6–7.5 months [4]. In comparison, 60.2% of patients were decannulated in the present investigation during an observational period of 31d after tracheostomy. A reason for the diverging findings may lie in the complex pathophysiology of dysphagia in neuro-critically ill patients. The neurological disease itself induces a central and/or peripheral lesion of the swallowing network [37, 49] and the sequelae of the ICU treatment itself can add to or even cause OD. Thus, apart from critical illness polyneuropathy, edema and local inflammation of the mucosa following the insertion of tubes along the pharynx as well as sedating medication can cause sensory impairment [25]. Impaired sensorium, however, is linked with an increased risk of aspiration [28, 30] whereas improved pharyngeal sensory feedback is related to improved swallowing function and a higher probability of (early) decannulation [43].

Age is another important contributing factor to OD in the neuro-critically ill. In the present investigation, higher age was associated with longer periods of the patients staying cannulated, which has also been described in other collectives [3, 33, 36]. Apart from the detrimental effect of age on the peripheral sensory system with the afore-mentioned consequences, increasing age is also linked to decreased muscle mass and function and reduced cortical plasticity, all of which critically contribute to impaired deglutition [40, 48].

Finally, the recent results suggest that the initial score of the SESETD may be predictive of decannulation probability during the course of treatment in the ICU, thereby corroborating findings derived from a cohort of GBS patients [37]. Hence, the initial score may aid in guiding decannulation decision in everyday clinical care, particularly considering optimal periods between first FEES and follow-up examinations in the future.

Strengths of this study were its prospective design, the objective dysphagia assessment performed by experienced endoscopists and the inclusion of a heterogeneous patient cohort in an acute setting. Several limitations to this study also need to be mentioned. First, since patients were only closely followed during their stay in the acute care facility there was no long-term follow-up. Second, since this was a single-center study, the results may be influenced by local standards of care and therefore not necessarily be transferable to other settings. Third, age was included in the regression analysis despite not being a significant factor in the univariate analysis. It needs to be considered that there are indications that age is not a good surrogate marker for comorbidities and frailty, however we intended to include a rather general factor that can easily be identified in the clinical setting since the protocol is designed for everyday use in the neuro-ICU. Fourth, in the present study stroke was comparatively overrepresented whereas other diseases were much rarer. It therefor needs to be considered that the generalizability of findings is limited, particularly in patients suffering from GBS, myopathy or meningitis.

## Conclusion

The recent findings suggest that the SESETD is a safe, objective and easy to use bedside tool to guide decannulation decisions in the neuro-critically ill. The SESETD may aid in predicting the likelihood of decannulation during the stay in the ICU.

## Supplementary Information

**Additional file 1: Supplement Figure 1.** Kaplan-Meier-Curve on time from decannulation to reintubation due to respiratory failure / dysphagia.

**Additional file 2: Supplement Figure 2.** Receiver operator characteristics curve: successful decannulation during course of stay depending on the initial score of the SESETD. Area under the curve: 0.678 [95%-CI: 0.623–0.732]; Sensitivity: 0.64; Specificity: 0.66; Positive Predictive Value: 0.74; Negative Predictive Value: 0.45.

## Data Availability

The datasets generated and/or analysed during the current study are available from the corresponding author on reasonable request.

## Abbreviations

DF: Decannulation failure
FEES: Flexible endoscopic evaluation of swallowing
FOIS: Functional oral intake scale
ICU: Intensive care unit
LOS: Length of stay
mRS: Modified Rankin Scale
MV: Mechanical ventilation
OD: Oropharyngeal dysphagia
SESETD: Standardized Endoscopic Swallowing Evaluation for Tracheostomy Decannulation in Critically Ill Neurologic Patients
TT: Tracheostomy tube
