# Supplementary figures and images for: Standardized Endoscopic Swallowing Evaluation for Tracheostomy Decannulation in Critically Ill Neurologic Patients – a prospective evaluation
Source: Neurol Res Pract. 2021 May 10;3:26. doi: 10.1186/s42466-021-00124-1 (PMC8108459; doi:10.1186/s42466-021-00124-1)

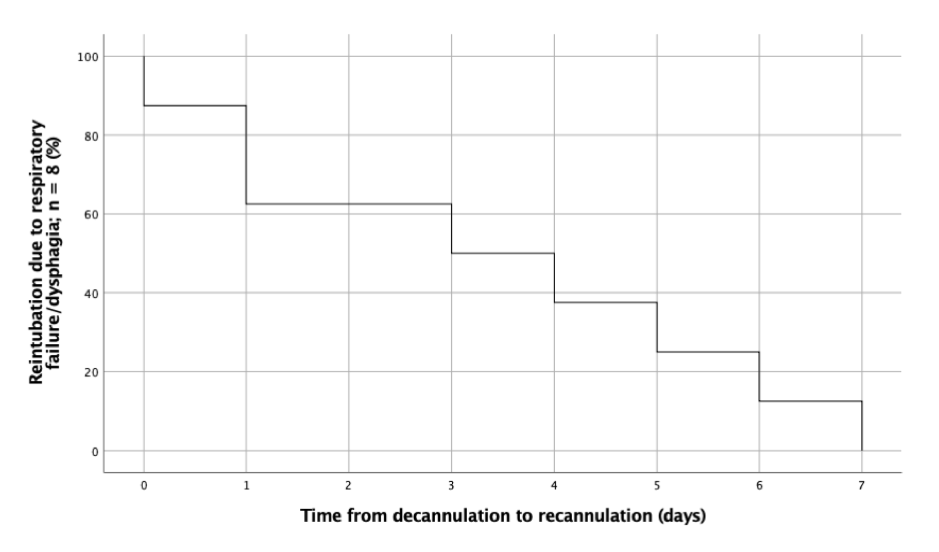

Supplement: Supplementary file 1 — Additional file 1: Supplement Figure 1. Kaplan-Meier-Curve on time from decannulation to reintubation due to respiratory failure / dysphagia. [file 42466_2021_124_MOESM1_ESM.tiff]

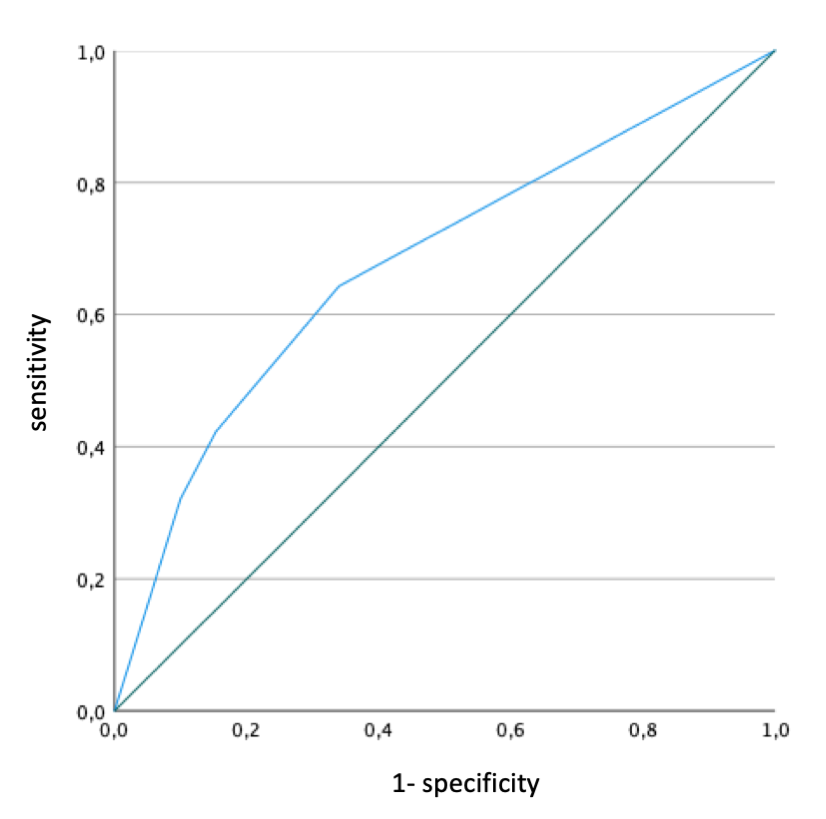

Supplement: Supplementary file 2 — Additional file 2: Supplement Figure 2. Receiver operator characteristics curve: successful decannulation during course of stay depending on the initial score of the SESETD. Area under the curve: 0.678 [95%-CI: 0.623–0.732]; Sensitivity: 0.64; Specificity: 0.66; Positive Predictive Value: 0.74; Negative Predictive Value: 0.45. [file 42466_2021_124_MOESM2_ESM.tiff]
